# Supplementary material for: Efficacy, Safety, and Immunogenicity of Subunit Respiratory Syncytial Virus Vaccines: Systematic Review and Meta-Analysis of Randomized Controlled Trials
Source: Vaccines (Basel). 2024 Aug 2;12(8):879. doi: 10.3390/vaccines12080879 (PMC11360664; doi:10.3390/vaccines12080879)
Supplement: Supplementary file 1 [file vaccines-12-00879-s001.zip › vaccines-3094600-supplementary.pdf]

Supplementary Materials

Table S1. Search strategy (Pubmed)

#	Query (P+I)	Results
1	"Respiratory Syncytial Viruses"[Mesh]	10893
2	Respiratory Syncytial Virus OR Syncytial Virus, Respiratory OR Virus, Respiratory Syncytial OR RSV Respiratory Syncytial Virus OR Orthopneumovirus OR Orthopneumoviruses OR Chimpanzee Coryza Agent OR Chimpanzee Coryza Agents OR Coryza Agent, Chimpanzee	19498
3	1 OR 2	19498
4	"Respiratory Tract Infections"[Mesh]	637495
5	Infection, Respiratory Tract OR Respiratory Tract Infection OR Infections, Respiratory OR Infections, Respiratory Tract OR Respiratory System Infections OR Infection, Respiratory System OR Respiratory System Infection OR Respiratory Infections OR Upper Respiratory Tract Infections OR Upper Respiratory Infections OR Upper Respiratory Tract Infection OR Infections, Upper Respiratory Tract OR Infections, Upper Respiratory OR Respiratory Infection, Upper	719098
6	4 OR 5	719098
7	3 OR 6	722537
8	"Vaccines"[Mesh]	283095
9	Vaccine	517907
10	8 OR 9	517907
11	"Vaccination"[Mesh]	112328
12	Vaccinations OR Immunization, Active OR Active Immunization OR Active Immunizations OR Immunizations, Active	522934
13	11 OR 12	522934
14	"Vaccines, Subunit"[Mesh]	8536
15	Subunit Vaccines OR Subunit Vaccine OR Vaccine, Subunit	18918
16	14 OR 15	18918
17	10 OR 13 OR 16	522934
18	"Randomized Controlled Trials as Topic"[Mesh]	169937
19	Clinical Trials, Randomized OR Trials, Randomized Clinical OR Controlled Clinical Trials, Randomized	769679
20	18 OR 19	769679
21	7 AND 17 AND 20	3229

Table S2 Studies excluded based on full text.

Reference	Reason for exclusion
Ison, Michael & Papi, A & Langley, Joanne & Lee, D & Leroux-Roels, Isabel & Martinón-Torres, F & Schwarz, T & Zyl-smit, R & Dezutter, N & Schrevel, N & Fissette, L & David, Marie-Pierre & Wielen, M & Kostanyan, L & Hulstrøm, V. (2023). A Respiratory Syncytial Virus (RSV) Prefusion F Candidate Vaccine (RSVPreF3 OA) is Efficacious in Adults≥60 Years of Age (YOA). *Pneumologie*. 77.	Abstracts, comments
Schwarz, T & Hwang, S & Ylisastigui, P & Liu, C & Takazawa, K & Yono, M & Ervin, J & Andrews, Charles & Fogarty, Charles & Eckermann, T & Collete, D & Heusch, M & Schrevel, N & Salaun, Bruno & Lievens, M & Maréchal, C & Nakanwagi, P & Hulstrøm, V. (2023). A Candidate Respiratory Syncytial Virus (RSV) Prefusion F Protein Investigational Vaccine (RSVPreF3 OA) Is Immunogenic when Administered in Adults≥60 Years of Age: Results at 6 Months after Vaccination. *Pneumologie*. 77.	Abstracts, comments
Awar M, Mylonakis E. In older adults, an $AS01_E$-adjuvanted RSVPreF3 OA vaccine reduced RSV-related lower respiratory tract disease [published correction appears in Ann Intern Med. 2023 Dec;176(12):JC144. doi: 10.7326/J23-0087]. *Ann Intern Med*. 2023;176(6):JC62.	Abstracts, comments
Ison, Michael & Papi, Alberto & Langley, Joanne & Lee, Dong-Dun & Leroux-Roels, Isabel & Martinon-Torres, Federico & Schwarz, Tino & Zyl-Smit, Richard & Dezutter, Nancy & Schrevel, Nathalie & Fissette, Laurence & David, Marie-Pierre & Wielen, Marie & Kostanyan, Lusine & Hulstrøm, Veronica. (2023). A Respiratory Syncytial Virus (RSV) Prefusion F Protein Candidate Vaccine (RSVPreF3-OA) is Efficacious in Adults ≥ 60 Years of Age (YOA). *Diabetologie und Stoffwechsel*. 18.	Abstracts, comments
Michael G Ison, Alberto Papi, Joanne M Langley, Dong-Gun Lee, Isabel Leroux-Roels, Federico Martinon-Torres, Tino F Schwarz, Richard N Van Zyl-Smit, Nancy Dezutter, Nathalie De Schrevel, Laurence Fissette, Marie-Pierre David, Marie Van Der Wielen, Lusine Kostanyan, Veronica Hulstrøm, LB745. Respiratory Syncytial Virus (RSV) Prefusion F Protein Candidate Vaccine (RSVPreF3 OA) is Efficacious in Adults ≥ 60 Years of Age (YOA), *Open Forum Infectious Diseases*, Volume 9, Issue Supplement_2, December 2022, ofac492.1868.	Abstracts, comments
Tino F Schwarz, Shinn-Jang Hwang, Pedro P Ylisastigui, Chiu-Shong Liu, Kenji Takazawa, Makoto Yono, John E Ervin, Charles Andrews, Charles Fogarty, Tamara Eckermann, Delphine Collete, Magali de Heusch, Nathalie De Schrevel, Bruno Salaun, Marc Lievens, Céline Maréchal, Phoebe Nakanwagi, Veronica Hulstrøm, 96. A Candidate Respiratory Syncytial Virus (RSV) Prefusion F Protein Investigational Vaccine (RSVPreF3 OA) Is Immunogenic when Administered in Adults ≥ 60 Years of Age: Results at 6 Months after Vaccination, *Open Forum Infectious Diseases*, Volume 9, Issue Supplement_2, December 2022, ofac492.174.	Abstracts, comments
Tino F Schwarz, Shinn-Jang Hwang, Pedro P Ylisastigui, Chiu-Shong Liu, Kenji Takazawa, Makoto Yono, John E Ervin, Charles Andrews, Charles Fogarty, Tamara Eckermann, Miguel Vicco, Marc Lievens, Céline Maréchal, Phoebe Nakanwagi, Veronica Hulstrøm, 119. Safety and Reactogenicity of an Investigational Respiratory Syncytial Virus (RSV) Prefusion F Protein Vaccine for Adults ≥ 60 Years of Age (RSVPreF3 OA): an Interim Analysis at 6 Months after Vaccination, *Open Forum Infectious Diseases*, Volume 9, Issue Supplement_2, December 2022, ofac492.197.	Abstracts, comments
Edward E Walsh, Fernando Polack, Agnieszka Zareba, Ann R Falsey, Gonzalo Perez Marc, Qin Jiang, Kathy Schneider, David Cooper, Maria Maddalena Lino, Annaliesa S Anderson, Katherin U Jensen, Kena A Swanson, Alejandra C Gurtman, William C Gruber, Beate Schmoele-Thoma, LB748. Efficacy And Safety Of Bivalent	Abstracts, comments

Respiratory Syncytial Virus (RSVpreF) Vaccine In Older Adults, *Open Forum Infectious Diseases*, Volume 9, Issue Supplement_2, December 2022, ofac492.1871.	
Guiñazú, J. R., Tica, J., Andrews, C. P., Davis, M. G., De Smedt, P., Essink, B., ... & Mesaros, N. (2020, October). 121. A respiratory syncytial virus prefusion F protein (RSVPreF3) candidate vaccine administered in older adults in a Phase I/II randomized clinical trial is immunogenic. In *Open Forum Infectious Diseases* (Vol. 7, No. Supplement_1, pp. S188-S189). US: Oxford University Press.	Abstracts, comments
Tino Schwarz, Casey Johnson, Christine Grigat, Dan Apter, Peter Csonka, Niklas Lindblad, Thi Lien-Anh Nguyen, Feng F Gao, Jyoti Soni, Antonella Nadia Tullio, Ilse Dieussaert, Marta Picciolato, Ouzama Henry, 1239. Different Dose Levels of a Respiratory Syncytial Virus Maternal Vaccine Candidate (RSVPreF3) Administered to Non-pregnant Women in a Randomized Clinical Trial Are Immunogenic and Well Tolerated, *Open Forum Infectious Diseases*, Volume 7, Issue Supplement_1, October 2020, Pages S638–S639.	Abstracts, comments
Schmoele-Thoma, B., Falsey, A.R., Walsh, E.E., Swanson, K.A., Zareba, A.M., Cooper, D., Gruber, W.C., Jansen, K.U., Radley, D.R., Scott, D.A., & Dormitzer, P.R. (2019). 2755. Phase 1/2, First-in-Human Study of the Safety, Tolerability, and Immunogenicity of an RSV Prefusion F-Based Subunit Vaccine Candidate. *Open Forum Infectious Diseases, 6*, S970 - S970.	Abstracts, comments
Vlachantoni, I.T., Ascough, S., Grimaldi, R., Leenhouts, K.J., Chiu, C., & Openshaw, P.J. (2017). S68 Phase 1 trial of an intranasal respiratory syncytial virus (rsv) subunit candidate vaccine: safety results from the muc-syngem study. *Thorax, 72*, A43 - A44.	Abstracts, comments
Matte, P., Feldman, R., Incalzi, R. A., Steenackers, K., Lee, D., Fissette, L., ... & Hulstrøm, V. (2023). EFFICACY OF A RESPIRATORY SYNCYTIAL VIRUS (RSV) PREFUSION F PROTEIN VACCINE (RSVPREF3 OA) IN OLDER ADULTS WITH PRE-EXISTING MEDICAL CONDITIONS. *Canadian Journal of Diabetes*, *47*(7), S160-S161.	Abstracts, comments
Wesley C, Winckworth LC. Respiratory syncytial virus vaccination in pregnancy is not effective enough at reducing infant infections. Arch Dis Child Educ Pract Ed. 2022;107(5):389.	Unable to get the full text
Domachowske JB, Bianco V, Ceballos A, et al. 2855. Respiratory Syncytial Virus Neutralizing Antibodies in Cord Blood and Serum from Infants up to 2 Years of Age in a Multinational Prospective Study. *Open Forum Infect Dis*. 2019;6(Suppl 2):S74-S75. Published 2019 Oct 23.	Unable to get the full text
Efficacy of bivalent prefusion F vaccine during pregnancy in preventing respiratory syncytial virus-related diseases in infants: a phase III, double-blind, randomized controlled trial.	Unable to get the full text
Louis F Fries, Iksung Cho, D Nigel Thomas, Judy L Wen, Michelle S Spindler, Amy B Fix, Joyce S Plested, Chang-Min Chen, Gregory M Glenn, 2637. Third Trimester Immunization with an Respiratory Syncytial Virus F Protein Vaccine for the Prevention of RSV Lower Respiratory Tract Infection in Infants, *Open Forum Infectious Diseases*, Volume 6, Issue Supplement_2, October 2019, Pages S921–S922.	Unable to get the full text
Shinde V, Cho I, Thomas N, et al. Post-Hoc Analyses of a Phase 2 and Phase 3 Efficacy Trial of an Unadjuvanted Respiratory Syncytial Virus (RSV) F-Glycoprotein Vaccine in Older Adults in the US: A Consistent Signal of Efficacy Against Hospitalizations for Acute Exacerbation of COPD (AECOPD)[M]//American Thoracic Society, 2019:A7094.	Unable to get the full text
Joanne Langley, Lisa Macdonald, Genevieve Weir, Donna Mackinnon-Cameron, Lingyun Ye, Shelly McNeil, Bert Schepens, Xavier Saelens, Marianne Stanford, Scott Halperin, A Phase I Randomized, Observer-Blind, Controlled, Dose Escalation Trial of the Safety and Tolerability of Two Intramuscular Doses of DPX-RSV(A), a	Unable to get the full text

Respiratory Syncytial Virus (RSV) Vaccine Containing RSV SH Antigen and a Novel Adjuvant DepoVax, or SH Antigen Co-administered With Aluminum Hydroxide, or Placebo to Healthy Adults ≥50–64 Years of Age, *Open Forum Infectious Diseases*, Volume 3, Issue suppl_1, December 2016, 1270.	
Piedra PA, Grace S, Jewell A, et al. Sequential annual administration of purified fusion protein vaccine against respiratory syncytial virus in children with cystic fibrosis. *Pediatr Infect Dis J*. 1998;17(3):217-224.	Unable to get the full text
Piedra PA, Grace S, Jewell A, et al. Purified fusion protein vaccine protects against lower respiratory tract illness during respiratory syncytial virus season in children with cystic fibrosis. *Pediatr Infect Dis J*. 1996;15(1):23-31.	Unable to get the full text
Purified fusion protein (PFP-2) vaccine protects against lower respiratory tract illness during respiratory syncytial virus (RSV) season in children with cystic fibrosis (CF) | Cochrane Library	Unable to get the full text
Paradiso PR, Hildreth SW, Hogerman DA, et al. Safety and immunogenicity of a subunit respiratory syncytial virus vaccine in children 24 to 48 months old. *Pediatr Infect Dis J*. 1994;13(9):792-798.	Unable to get the full text
Welliver RC, Tristram DA, Batt K, Sun M, Hogerman D, Hildreth S. Respiratory syncytial virus-specific cell-mediated immune responses after vaccination with a purified fusion protein subunit vaccine. *J Infect Dis*. 1994;170(2):425-428.	Unable to get the full text
Tristram DA, Welliver RC, Mohar CK, Hogerman DA, Hildreth SW, Paradiso P. Immunogenicity and safety of respiratory syncytial virus subunit vaccine in seropositive children 18-36 months old. *J Infect Dis*. 1993;167(1):191-195.	Unable to get the full text
Madhi SA, Polack FP, Piedra PA, et al. Respiratory Syncytial Virus Vaccination during Pregnancy and Effects in Infants. *N Engl J Med*. 2020;383(5):426-439.	Not subunit vaccines
https://www.cochranelibrary.com/central/doi/10.1002/central/CN-02280685/full	Not RCTs
Yu J, Powers JH 3rd, Vallo D, Falloon J. Evaluation of Efficacy Endpoints for a Phase IIb Study of a Respiratory Syncytial Virus Vaccine in Older Adults Using Patient-Reported Outcomes With Laboratory Confirmation. *Value Health*. 2020;23(2):227-235.	Not RCTs
Chu HY, Katz J, Tielsch J, et al. Clinical Presentation and Birth Outcomes Associated with Respiratory Syncytial Virus Infection in Pregnancy. *PLoS One*. 2016;11(3):e0152015. Published 2016 Mar 31.	Not RCTs
Ascough S, Vlachantoni I, Kalyan M, et al. Local and Systemic Immunity against Respiratory Syncytial Virus Induced by a Novel Intranasal Vaccine. A Randomized, Double-Blind, Placebo-controlled Clinical Trial. *Am J Respir Crit Care Med*. 2019;200(4):481-492.	No data available
Crank MC, Ruckwardt TJ, Chen M, et al. A proof of concept for structure-based vaccine design targeting RSV in humans. *Science*. 2019;365(6452):505-509.	No data available
Weinberg A, Lambert SL, Canniff J, et al. Antibody and B cell responses to an investigational adjuvanted RSV vaccine for older adults. *Hum Vaccin Immunother*. 2019;15(10):2466-2474.	No data available
Munoz FM, Piedra PA, Glezen WP. Safety and immunogenicity of respiratory syncytial virus purified fusion protein-2 vaccine in pregnant women. *Vaccine*. 2003;21(24):3465-3467.	No data available
Power UF, Nguyen TN, Rietveld E, et al. Safety and immunogenicity of a novel recombinant subunit respiratory syncytial virus vaccine (BBG2Na) in healthy young adults. *J Infect Dis*. 2001;184(11):1456-1460.	No data available
Falsey AR, Walsh EE. Safety and immunogenicity of a respiratory syncytial virus subunit vaccine (PFP-2) in ambulatory adults over age 60. *Vaccine*. 1996;14(13):1214-1218.	No data available

Feldman RG, Antonelli-Incalzi R, Steenackers K, et al. Respiratory Syncytial Virus Prefusion F Protein Vaccine Is Efficacious in Older Adults With Underlying Medical Conditions. *Clin Infect Dis*. 2024;78(1):202-209.	Duplicate publication
ROBERT G. FELDMAN, RAFFAELE ANTONELLI INCALZI, KATIE STEENACKERS, DONG-GUN LEE, LAURENCE FISSETTE, MARIE-PIERRE DAVID, CÉLINE MARÉCHAL, LUSINE KOSTANYAN, MARIE VAN DER WIELEN, VERONICA HULSTROEM; 280-LB: Efficacy of a Respiratory Syncytial Virus (RSV) Prefusion F Protein Vaccine (RSVPreF3 OA) in Older Adults with Preexisting Medical Conditions. *Diabetes* 20 June 2023; 72 (Supplement_1): 280–LB.	Duplicate publication
Chandler R, Montenegro N, Llorach C, et al. Immunogenicity, Reactogenicity, and Safety of AS01E-adjuvanted RSV Prefusion F Protein-based Candidate Vaccine (RSVPreF3 OA) When Co-administered With a Seasonal Quadrivalent Influenza Vaccine in Older Adults: Results of a Phase 3, Open-Label, Randomized Controlled Trial. *Clin Infect Dis*. Published online January 8, 2024.	Duplicate publication
Madhi SA, Polack FP, Piedra PA, et al. Respiratory Syncytial Virus Vaccination during Pregnancy and Effects in Infants. *N Engl J Med*. 2020;383(5):426-439.	Duplicate publication
Ruckwardt TJ, Morabito KM, Phung E, et al. Safety, tolerability, and immunogenicity of the respiratory syncytial virus prefusion F subunit vaccine DS-Cav1: a phase 1, randomised, open-label, dose-escalation clinical trial. *Lancet Respir Med*. 2021;9(10):1111-1120.	Control group were not placebo
Beran J, Lickliter JD, Schwarz TF, et al. Safety and Immunogenicity of 3 Formulations of an Investigational Respiratory Syncytial Virus Vaccine in Nonpregnant Women: Results From 2 Phase 2 Trials. *J Infect Dis*. 2018;217(10):1616-1625.	Control group were not placebo
Langley JM, Sales V, McGeer A, et al. A dose-ranging study of a subunit Respiratory Syncytial Virus subtype A vaccine with and without aluminum phosphate adjuvantation in adults > or =65 years of age. *Vaccine*. 2009;27(42):5913-5919.	Control group were not placebo
Piedra PA, Cron SG, Jewell A, et al. Immunogenicity of a new purified fusion protein vaccine to respiratory syncytial virus: a multi-center trial in children with cystic fibrosis. *Vaccine*. 2003;21(19-20):2448-2460.	Control group were not placebo
Piedra PA, Glezen WP, Kasel JA, et al. Safety and immunogenicity of the PFP vaccine against respiratory syncytial virus (RSV): the western blot assay aids in distinguishing immune responses of the PFP vaccine from RSV infection. *Vaccine*. 1995;13(12):1095-1101.	Control group were not placebo
Chandler R, Montenegro N, Llorach C, et al. Immunogenicity, Reactogenicity, and Safety of AS01E-adjuvanted RSV Prefusion F Protein-based Candidate Vaccine (RSVPreF3 OA) When Co-administered With a Seasonal Quadrivalent Influenza Vaccine in Older Adults: Results of a Phase 3, Open-Label, Randomized Controlled Trial. *Clin Infect Dis*. Published online January 8, 2024.	Combination of vaccines
Athan E, Baber J, Quan K, et al. Safety and Immunogenicity of Bivalent RSVpreF Vaccine Coadministered With Seasonal Inactivated Influenza Vaccine in Older Adults. *Clin Infect Dis*. 2024;78(5):1360-1368.	Combination of vaccines
Hermida N, Ferguson M, Leroux-Roels I, et al. Safety and Immunogenicity of Respiratory Syncytial Virus Pre-fusion Maternal Vaccine Co-administered with Diphtheria-Tetanus-Pertussis Vaccine: A Phase 2 Study. *J Infect Dis*. Published online December 22, 2023.	Combination of vaccines
Baber J, Arya M, Moodley Y, et al. A Phase 1/2 Study of a Respiratory Syncytial Virus Prefusion F Vaccine With and Without Adjuvant in Healthy Older Adults. *J Infect Dis*. 2022;226(12):2054-2063.	Combination of vaccines

Peterson JT, Zareba AM, Fitz-Patrick D, et al. Safety and Immunogenicity of a Respiratory Syncytial Virus Prefusion F Vaccine When Coadministered With a Tetanus, Diphtheria, and Acellular Pertussis Vaccine. *J Infect Dis*. 2022;225(12):2077-2086.	Combination of vaccines
Falloon J, Talbot HK, Curtis C, et al. Dose Selection for an Adjuvanted Respiratory Syncytial Virus F Protein Vaccine for Older Adults Based on Humoral and Cellular Immune Responses. *Clin Vaccine Immunol*. 2017;24(9):e00157-17.	Combination of vaccines
Falloon J, Yu J, Esser MT, et al. An Adjuvanted, Postfusion F Protein-Based Vaccine Did Not Prevent Respiratory Syncytial Virus Illness in Older Adults. *J Infect Dis*. 2017;216(11):1362-1370.	Combination of vaccines
Falsey AR, Walsh EE, Capellan J, et al. Comparison of the safety and immunogenicity of 2 respiratory syncytial virus (rsv) vaccines--nonadjuvanted vaccine or vaccine adjuvanted with alum--given concomitantly with influenza vaccine to high-risk elderly individuals. *J Infect Dis*. 2008;198(9):1317-1326.	Combination of vaccines
Gonzalez IM, Karron RA, Eichelberger M, et al. Evaluation of the live attenuated cpts 248/404 RSV vaccine in combination with a subunit RSV vaccine (PFP-2) in healthy young and older adults. *Vaccine*. 2000;18(17):1763-1772.	Combination of vaccines
Groothuis JR, King SJ, Hogerman DA, Paradiso PR, Simoes EA. Safety and immunogenicity of a purified F protein respiratory syncytial virus (PFP-2) vaccine in seropositive children with bronchopulmonary dysplasia. *J Infect Dis*. 1998;177(2):467-469.	Combination of vaccines

Table S3. Quality Assessment of included studies.

study	Randomization process	Deviations from intended interventions	Missing outcome data	Measurement of the outcome	Selection of the reported result	Overall Bias
**RSV-ARI**						
Papi A, 2023[27]	Low	Some concerns	Some concerns	Low	Low	Some concerns
Schmoele -Thoma B,2022[28]	Low	Low	Low	Low	Low	Low
Walsh EE,2023[33]	Some concerns	Low	Some concerns	Low	Low	Some concerns
**RSV-LRTI**						
Kampmann B,2023[21]	Some concerns	Some concerns	Low	Low	Low	Some concerns
Papi A, 2023[27]	Low	Some concerns	Some concerns	Low	Low	Some concerns
Simões EAF, 2022[31]	High	High	High	High	Some concerns	Some concerns
Walsh EE,2023[33]	Some concerns	Some concerns	Some concerns	Low	Low	Some concerns
**RSV-SLRTI**						
Kampmann B,2023[21]	Some concerns	Some concerns	Low	Low	Low	Some concerns
Papi A, 2023[27]	Low	Some concerns	Some concerns	Low	Low	Some concerns
Simões EAF, 2022[31]	High	High	High	High	Some concerns	Some concerns
**nAb GMT**						
Bebia Z,2023[17]	Low	Some concerns	Low	Low	Low	Some concerns
Falsey AR, 2022[20]	Low	Low	Low	Low	Low	Low
Kotb S,2023[22]	Low	Low	Low	Low	Low	Low
Langley JM, 2017[23]	Low	Low	Low	Low	Low	Low
Leroux-Roels G, 2019[25]	Some concerns	Low	Low	Low	Low	Some concerns
Leroux-Roels I,2023[26]	Low	High	Low	Low	Low	High
Papi A, 2023[27]	Low	Some concerns	Low	Low	Low	Some concerns
Schmoele-Thoma B,2022[28]	Low	Low	Low	Low	Low	Low
Schwarz TF,2022[29]	Low	Some concerns	Low	Low	Low	Some concerns

Schwarz TF, 2019[30]	High	High	High	High	Some concerns	Some concerns
Simões EAF, 2022[31]	Low	Some concerns	Low	Low	Low	Some concerns
Walsh EE,2022[32]	Low	Some concerns	Low	Low	Low	Some concerns
**SAE**						
Bebia Z,2023[17]	Low	Low	Low	Low	Low	Low
Falloon J,2016[19]	Low	Low	Low	Low	Low	Low
Falsey AR, 2022[20]	Low	Low	Low	Low	Low	Low
Kampmann B,2023[21]	Some concerns	Some concerns	Low	Low	Low	Some concerns
Kotb S,2023[22]	Low	Low	Low	Low	Low	Low
Leroux-Roels I,2023[26]	Low	Low	High	High	Low	High
Papi A, 2023[27]	Low	Low	Low	Low	Low	Low
Schwarz TF,2022[29]	Low	Low	Low	Low	Low	Low
Schwarz TF, 2019[30]	Low	Low	Low	Low	Low	Low
Simões EAF, 2022[31]	High	High	High	High	Some concerns	Some concerns
Walsh EE,2022[32]	Low	Low	Low	Low	Low	Low
Walsh EE,2023[33]	Some concerns	Low	Low	Low	Low	Some concerns

Table S4 Evaluation of the quality of evidence using GRADE system for outcomes.

Outcomes	Illustrative comparative risks* (95% CI)		Relative effect (95% CI)	No of Participants (studies)	Quality of the evidence (GRADE)	SMD	Comments
	Assumed risk	Corresponding risk					
	**Control**	**Efficacy**					
**RSV-ARI**<br>Follow-up: mean 6.8 months	**Study population**		**RR 0.31**<br>(0.23 to 0.43)	57636<br>(3 studies)	⊕⊕⊕⊝<br>**moderate**		downgraded by one level for risk of bias (two studies with a moderate risk)
	**6 per 1000**	**2 per 1000**(1 to 3)					
	**Moderate**						
	**8 per 1000**	**2 per 1000**(2 to 3)					
**RSV-LRTI**<br>Follow-up: mean 6.8 months	**Study population**		**RR 0.32**<br>(0.22 to 0.44)	65057<br>(4 studies)	⊕⊕⊕⊝<br>**moderate**		downgraded by one level for risk of bias (four studies with a moderate risk)
	**4 per 1000**	**1 per 1000**(1 to 2)					
	**Moderate**						
	**10 per 1000**	**3 per 1000**(2 to 4)					
**RSV-SLRTI**<br>Follow-up: mean 6.8 months	**Study population**		**RR 0.13**<br>(0.06 to 0.29)	32443<br>(3 studies)	⊕⊕⊕⊝<br>**moderate**		downgraded by one level for risk of bias (three studies with a moderate risk)
	**3 per 1000**	**0 per 1000**(0 to 1)					
	**Moderate**						
	**10 per 1000**	**1 per 1000**(1 to 3)					
**Serious**<br>Follow-up: 6-12 months	**Study population**		**RR 1.05**<br>(0.98 to 1.14)	75255<br>(15 studies)	⊕⊕⊕⊝<br>**moderate**		downgraded by one level for risk of bias (one study with a high risk; three studies with a moderate risk)
	**33 per 1000**	**35 per 1000**(32 to 38)					
	**Moderate**						
	**50 per 1000**	**52 per 1000**(49 to 57)					

**nAb GMT**<br>Follow-up: mean 1 months		The mean gmt in the intervention groups was<br>**2.92 standard deviations higher**<br>(2.42 to 3.42 higher)		3112<br>(11 studies)	⊕⊕⊖⊖<br>**low**	SMD 2.92<br>(2.42 to 3.42)	downgraded by one level for risk of bias (one study with a high risk and seven studies with a moderate risk), and downgraded one level for serious inconsistency (I^2=93.9%)

High certainty: We are very confident that the true effect lies close to that of the estimate of the effect.

Moderate certainty: We are moderately confident in the effect estimate: The true effect is likely to be close to the estimate of the effect, but there is a possibility that it is substantially different.

Low certainty: Our confidence in the effect estimate is limited: The true effect may be substantially different from the estimate of the effect.

Very low certainty: We have very little confidence in the effect estimate: The true effect is likely to be substantially different from the estimate of effect.

nAb = neutralizing antibody.

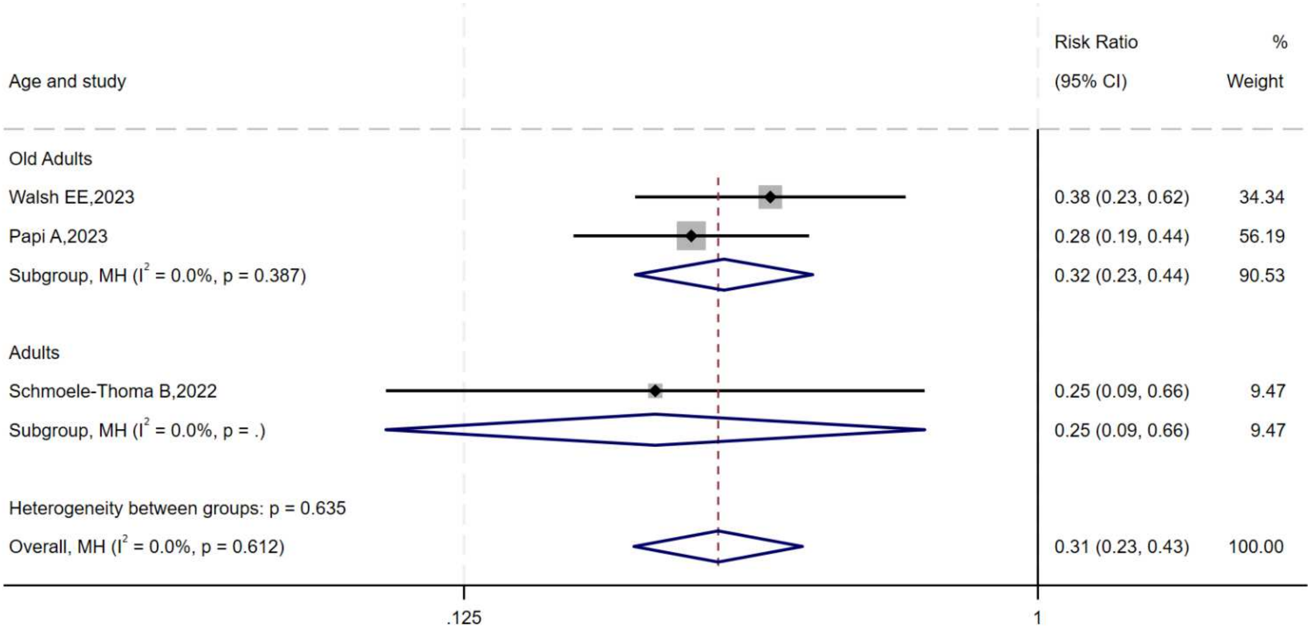
Age and study
Risk Ratio
(95% CI)
%
Weight
Old Adults
Walsh EE,2023
0.38 (0.23, 0.62)
34.34
Papi A,2023
0.28 (0.19, 0.44)
56.19
Subgroup, MH (I2 = 0.0%, p = 0.387)
0.32 (0.23, 0.44)
90.53
Adults
Schmoele-Thoma B,2022
0.25 (0.09, 0.66)
9.47
Subgroup, MH (I2 = 0.0%, p = .)
0.25 (0.09, 0.66)
9.47
Heterogeneity between groups: p = 0.635
Overall, MH (I2 = 0.0%, p = 0.612)
0.31 (0.23, 0.43)
100.00
.125
1

(**A**)

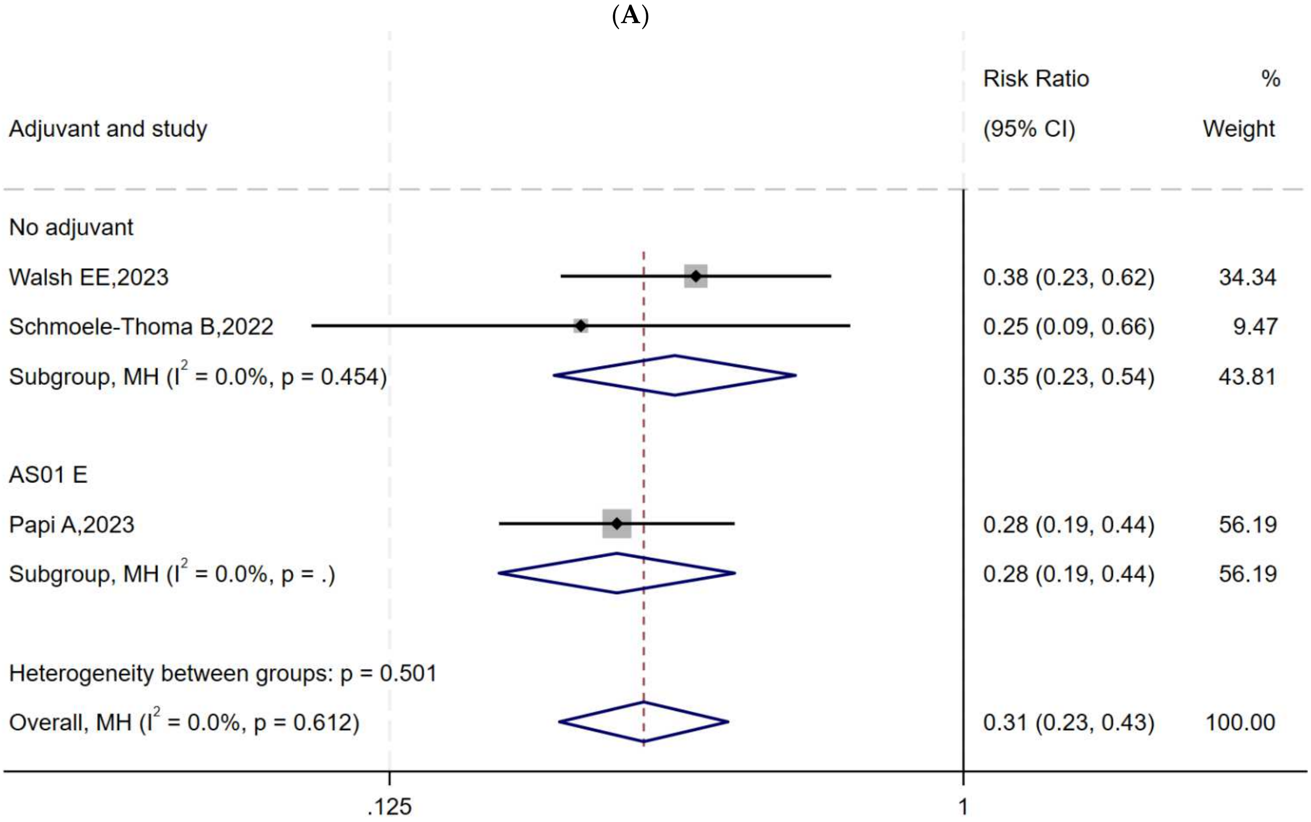
Adjuvant and study
Risk Ratio
(95% CI)
%
Weight
No adjuvant
Walsh EE,2023
0.38 (0.23, 0.62)
34.34
Schmoele-Thoma B,2022
0.25 (0.09, 0.66)
9.47
Subgroup, MH (I2 = 0.0%, p = 0.454)
0.35 (0.23, 0.54)
43.81
AS01 E
Papi A,2023
0.28 (0.19, 0.44)
56.19
Subgroup, MH (I2 = 0.0%, p = .)
0.28 (0.19, 0.44)
56.19
Heterogeneity between groups: p = 0.501
Overall, MH (I2 = 0.0%, p = 0.612)
0.31 (0.23, 0.43)
100.00
.125
1

(**B**)

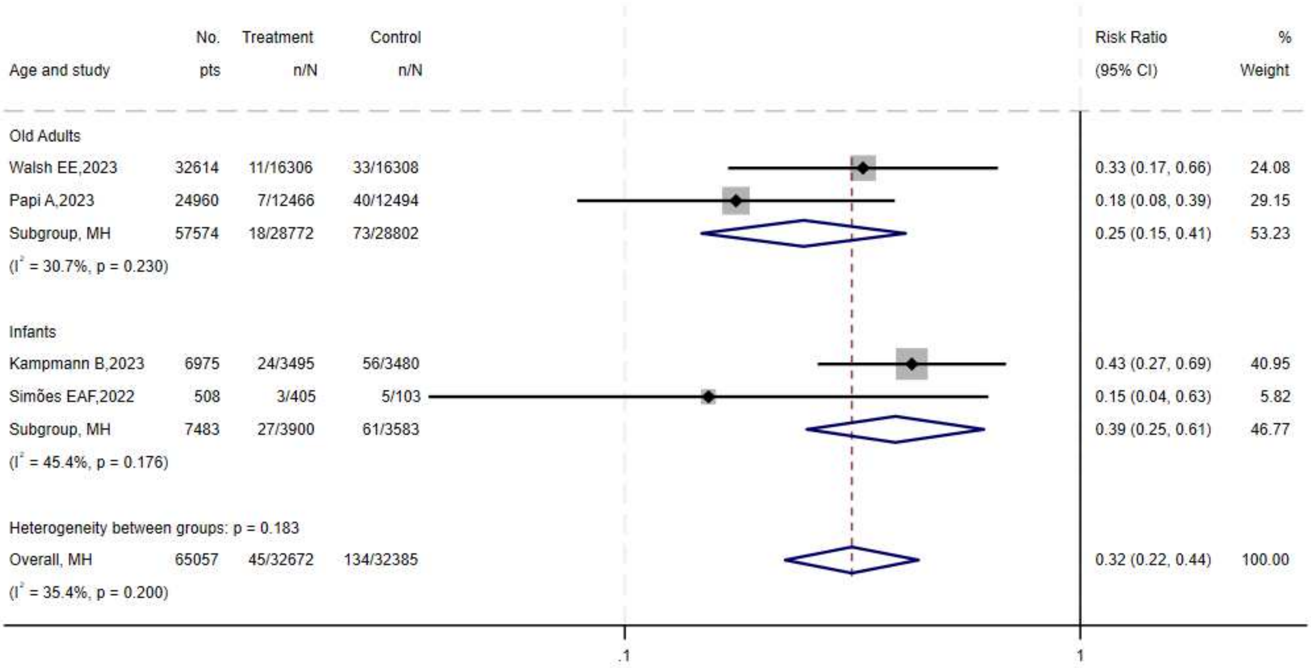
Age and study
No. pts
Treatment n/N
Control n/N
Risk Ratio (95% CI)
% Weight
Old Adults
Walsh EE,2023 32614 11/16306 33/16308 0.33 (0.17, 0.66) 24.08
Papi A,2023 24960 7/12466 40/12494 0.18 (0.08, 0.39) 29.15
Subgroup, MH 57574 18/28772 73/28802 0.25 (0.15, 0.41) 53.23
(I² = 30.7%, p = 0.230)
Infants
Kampmann B,2023 6975 24/3495 56/3480 0.43 (0.27, 0.69) 40.95
Simões EAF,2022 508 3/405 5/103 0.15 (0.04, 0.63) 5.82
Subgroup, MH 7483 27/3900 61/3583 0.39 (0.25, 0.61) 46.77
(I² = 45.4%, p = 0.176)
Heterogeneity between groups: p = 0.183
Overall, MH 65057 45/32672 134/32385 0.32 (0.22, 0.44) 100.00
(I² = 35.4%, p = 0.200)
.1
1

(**C**)

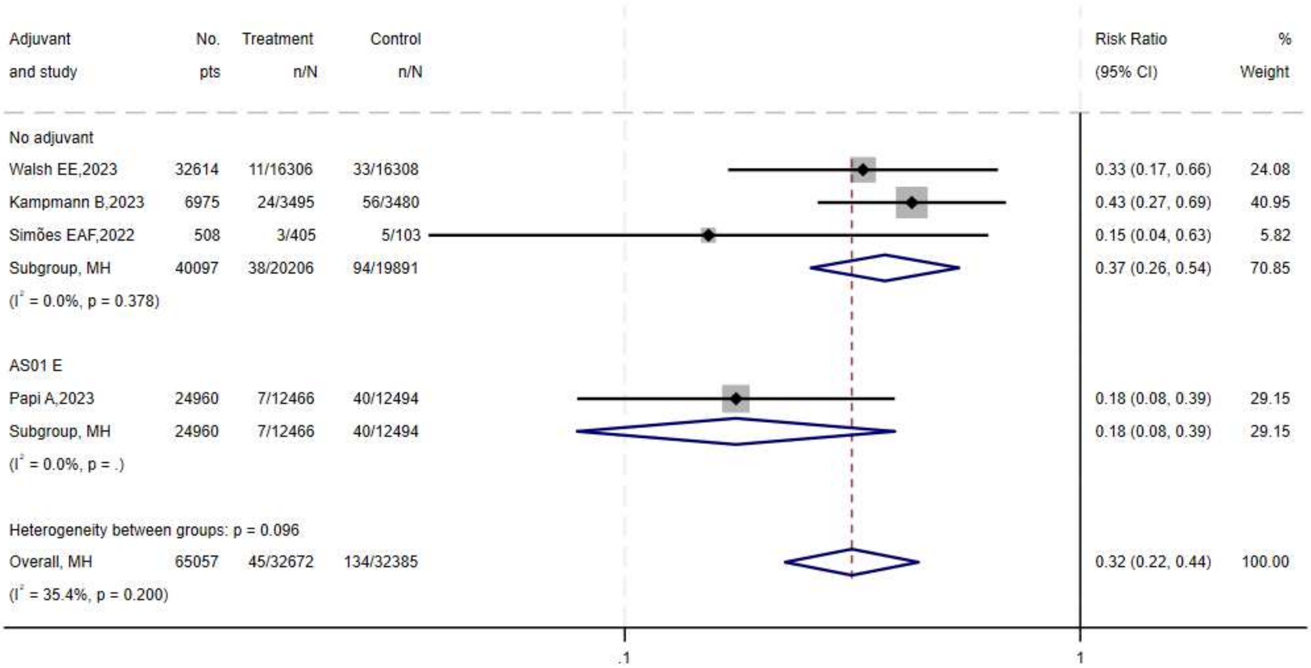
Adjuvant and study
No. pts
Treatment n/N
Control n/N
Risk Ratio (95% CI)
% Weight
No adjuvant
Walsh EE,2023 32614 11/16306 33/16308 0.33 (0.17, 0.66) 24.08
Kampmann B,2023 6975 24/3495 56/3480 0.43 (0.27, 0.69) 40.95
Simões EAF,2022 508 3/405 5/103 0.15 (0.04, 0.63) 5.82
Subgroup, MH 40097 38/20206 94/19891 0.37 (0.26, 0.54) 70.85
(I² = 0.0%, p = 0.378)
AS01 E
Papi A,2023 24960 7/12466 40/12494 0.18 (0.08, 0.39) 29.15
Subgroup, MH 24960 7/12466 40/12494 0.18 (0.08, 0.39) 29.15
(I² = 0.0%, p = .)
Heterogeneity between groups: p = 0.096
Overall, MH 65057 45/32672 134/32385 0.32 (0.22, 0.44) 100.00
(I² = 35.4%, p = 0.200)
.1
1

(**D**)

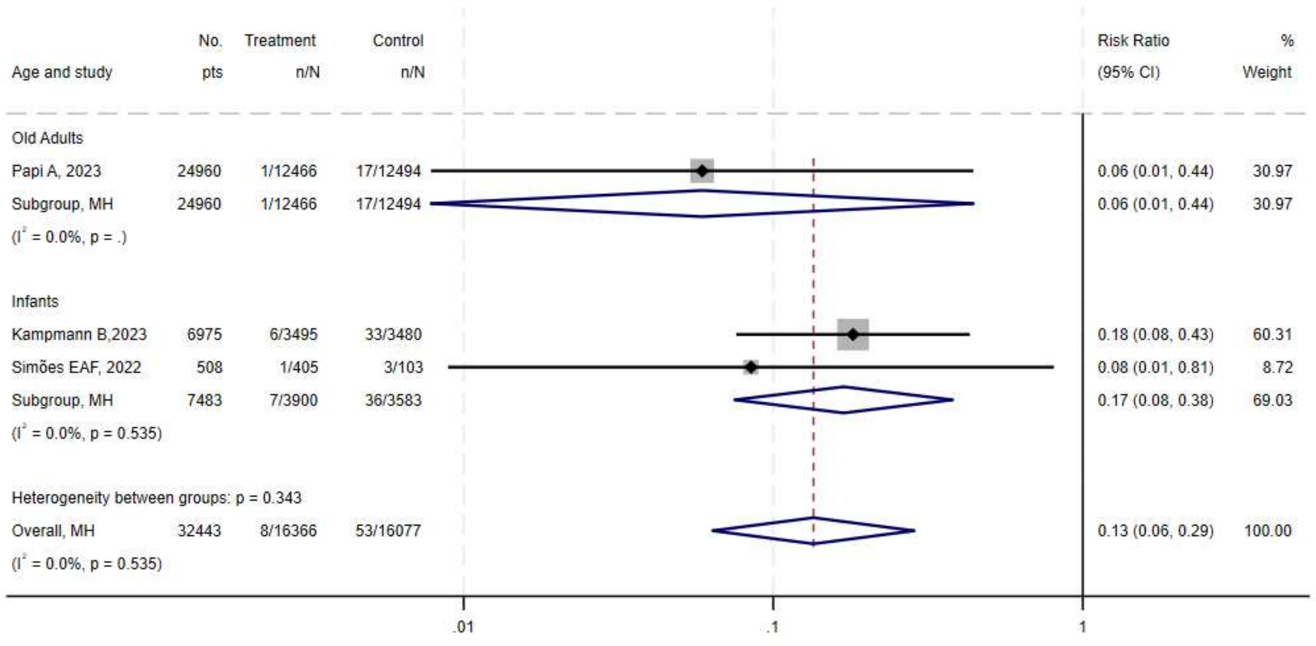

(**E**)

Figure S1. (**A**) Subgroup analysis for RSV-ARI by age. (**B**) Subgroup analysis for RSV-ARI by adjuvant. (**C**) Subgroup analysis for RSV-LRTI by age. (**D**) Subgroup analysis for RSV-LRTI by adjuvant. (**E**) Subgroup analysis for RSV-SLRTI by age. [21,27,28,31,33].

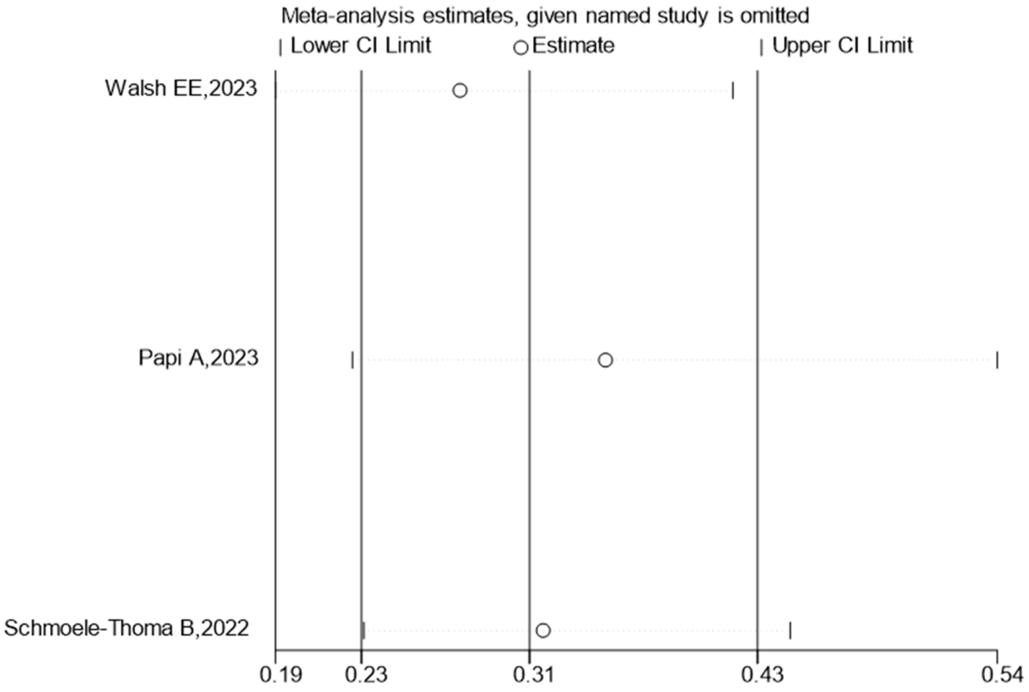
Meta-analysis estimates, given named study is omitted
Lower CI Limit
Estimate
Upper CI Limit
Walsh EE,2023
Papi A,2023
Schmoele-Thoma B,2022
0.19
0.23
0.31
0.43
0.54

(A)

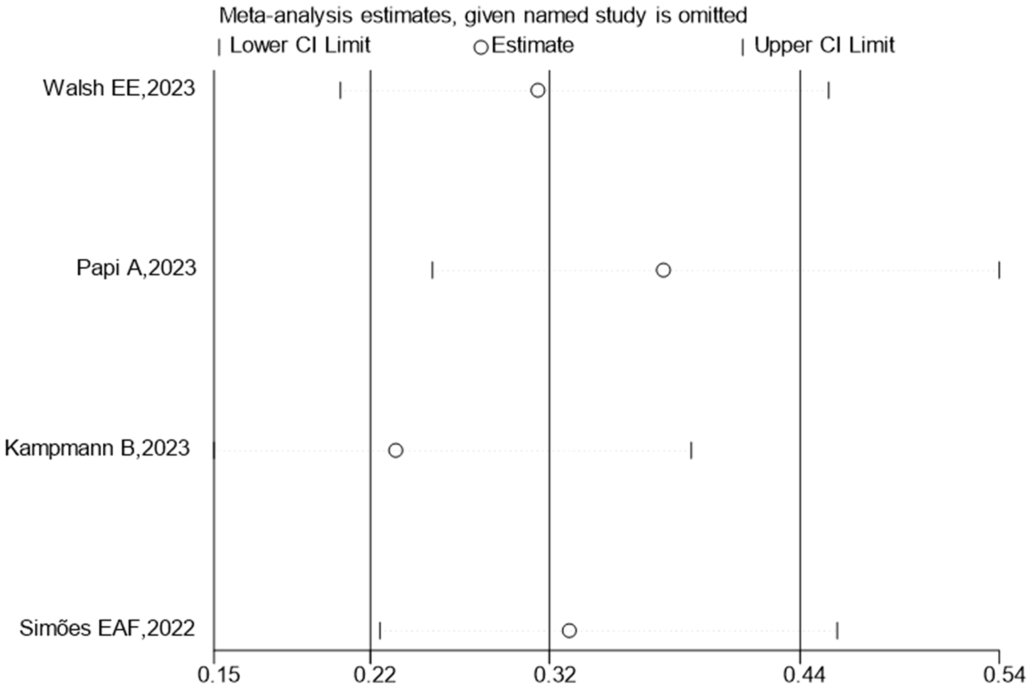
Meta-analysis estimates, given named study is omitted
Lower CI Limit
Estimate
Upper CI Limit
Walsh EE,2023
Papi A,2023
Kampmann B,2023
Simões EAF,2022
0.15
0.22
0.32
0.44
0.54

(B)

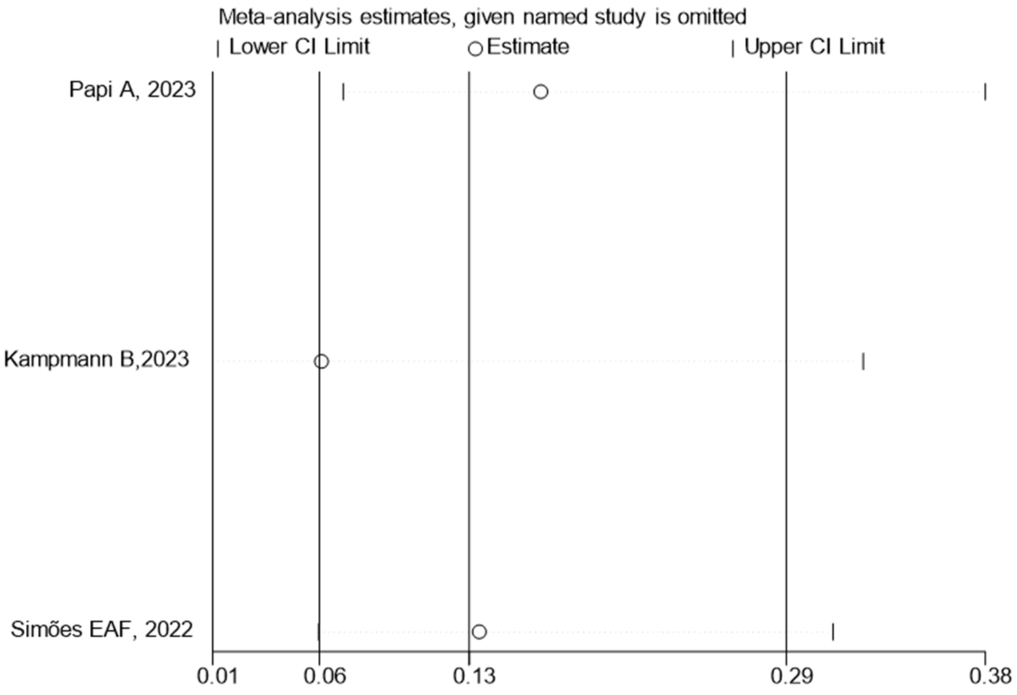

(C)

Figure S2. (**A**) Sensitivity analysis for RSV-ARI. (**B**) Sensitivity analysis for RSV-LRTI. (**C**) Sensitivity analysis for RSV-SLRTI. [21,27,28,31,33]

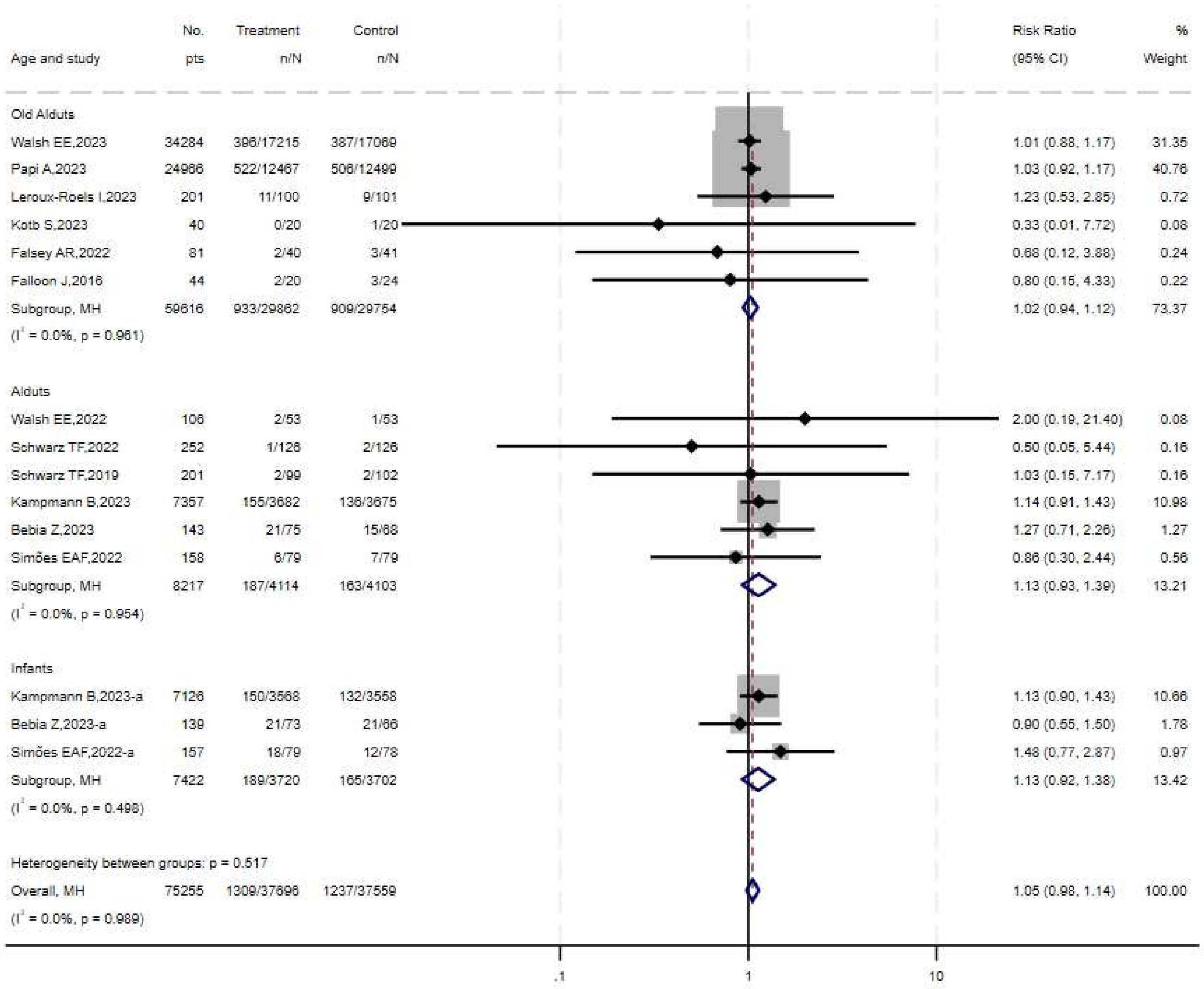
Age and study
No. pts
Treatment n/N
Control n/N
Risk Ratio (95% CI)
% Weight
Old Alduts
Walsh EE,2023 34284 396/17215 387/17069 1.01 (0.88, 1.17) 31.35
Papi A,2023 24966 522/12467 506/12499 1.03 (0.92, 1.17) 40.76
Leroux-Roels I,2023 201 11/100 9/101 1.23 (0.53, 2.85) 0.72
Kotb S,2023 40 0/20 1/20 0.33 (0.01, 7.72) 0.08
Falsey AR,2022 81 2/40 3/41 0.68 (0.12, 3.88) 0.24
Falloon J,2016 44 2/20 3/24 0.80 (0.15, 4.33) 0.22
Subgroup, MH 59616 933/29862 909/29754 1.02 (0.94, 1.12) 73.37
(I² = 0.0%, p = 0.961)
Alduts
Walsh EE,2022 106 2/53 1/53 2.00 (0.19, 21.40) 0.08
Schwarz TF,2022 252 1/126 2/126 0.50 (0.05, 5.44) 0.16
Schwarz TF,2019 201 2/99 2/102 1.03 (0.15, 7.17) 0.16
Kampmann B,2023 7357 155/3682 136/3675 1.14 (0.91, 1.43) 10.98
Bebia Z,2023 143 21/75 15/68 1.27 (0.71, 2.26) 1.27
Simões EAF,2022 158 6/79 7/79 0.86 (0.30, 2.44) 0.56
Subgroup, MH 8217 187/4114 163/4103 1.13 (0.93, 1.39) 13.21
(I² = 0.0%, p = 0.954)
Infants
Kampmann B,2023-a 7126 150/3568 132/3558 1.13 (0.90, 1.43) 10.66
Bebia Z,2023-a 139 21/73 21/66 0.90 (0.55, 1.50) 1.78
Simões EAF,2022-a 157 18/79 12/78 1.48 (0.77, 2.87) 0.97
Subgroup, MH 7422 189/3720 165/3702 1.13 (0.92, 1.38) 13.42
(I² = 0.0%, p = 0.498)
Heterogeneity between groups: p = 0.517
Overall, MH 75255 1309/37696 1237/37559 1.05 (0.98, 1.14) 100.00
(I² = 0.0%, p = 0.989)
.1
1
10

(A)

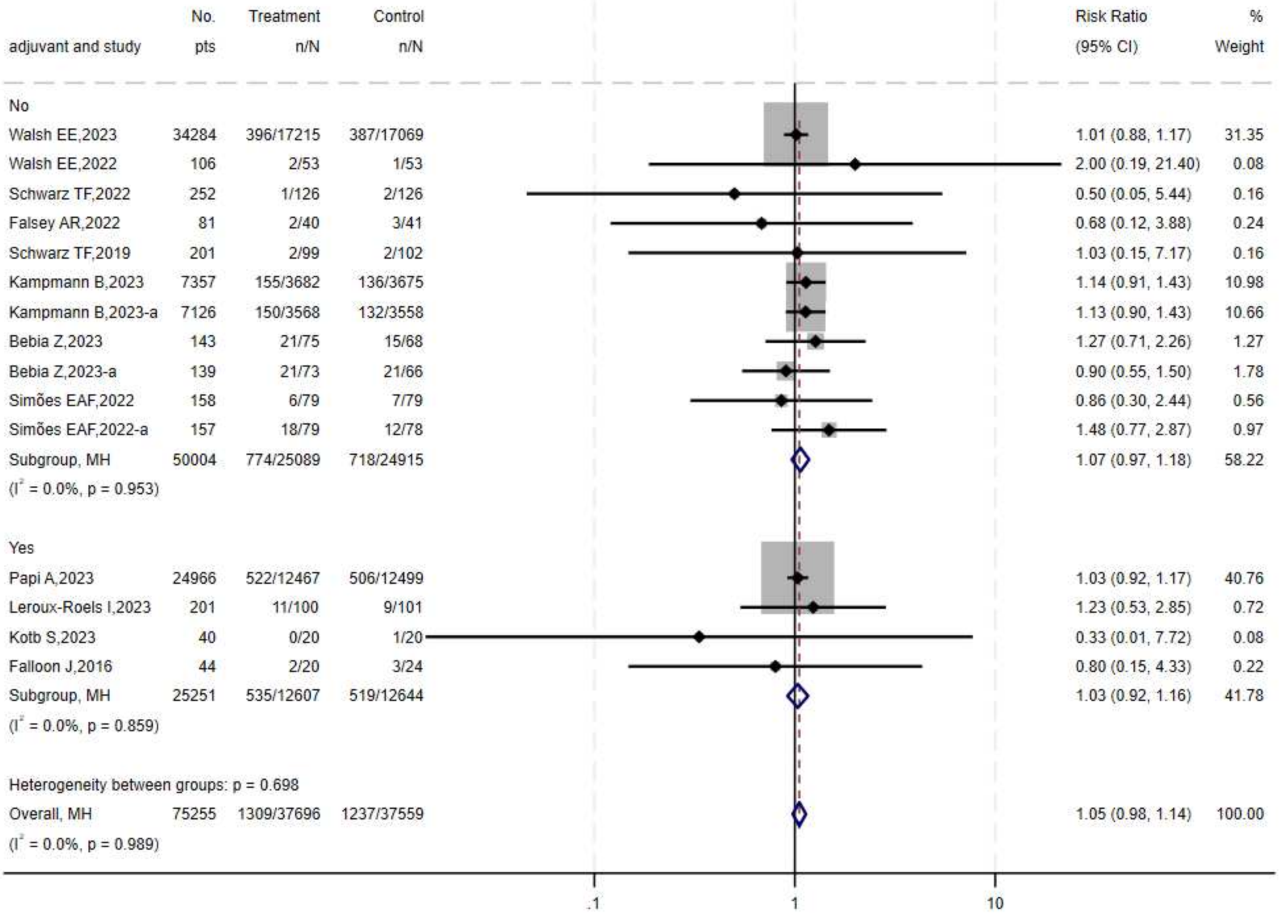

(B)

Figure S3. (**A**) Subgroup analysis for SAE by age. (**B**). Subgroup analysis for SAE by adjuvant. [17,19-22,26,27,29-33].

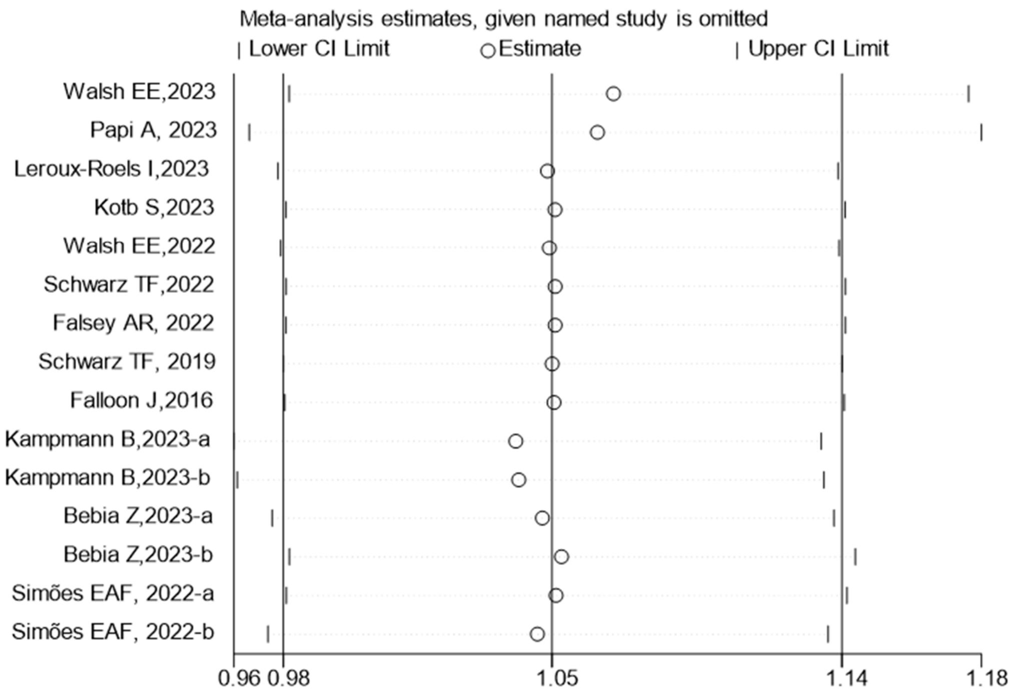

Figure S4. Sensitivity analysis for SAE. [17,19-22,26,27,29-33].

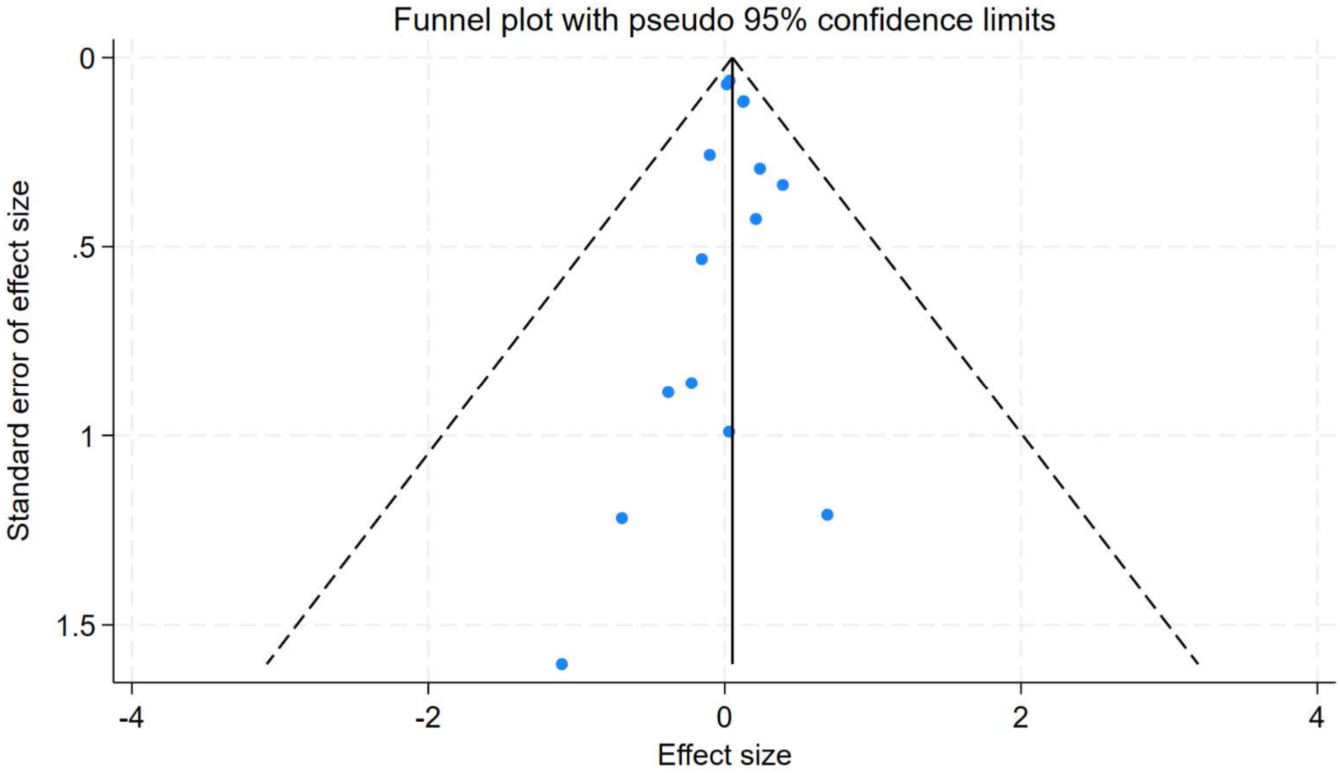

Figure S5. Funnel plot of SAE. [17,19-22,26,27,29-33].

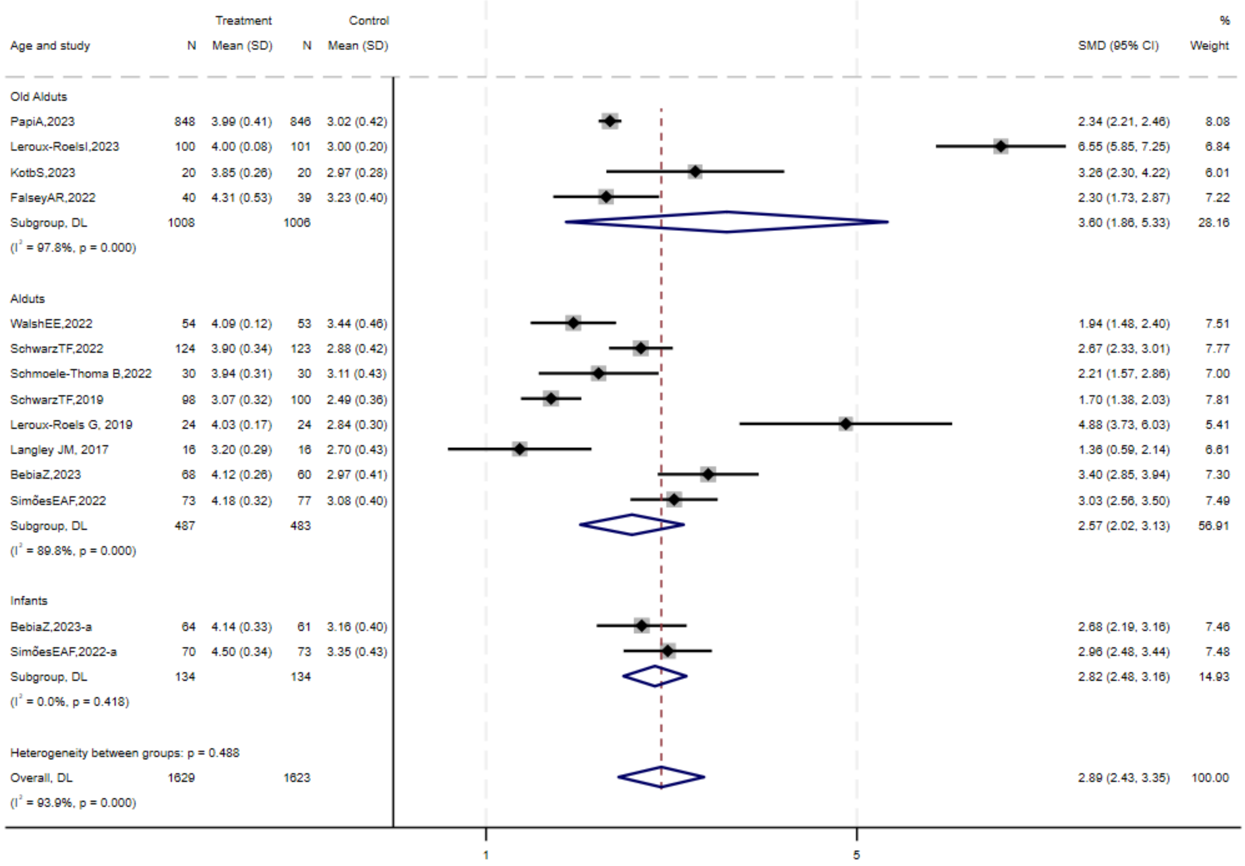

(A)

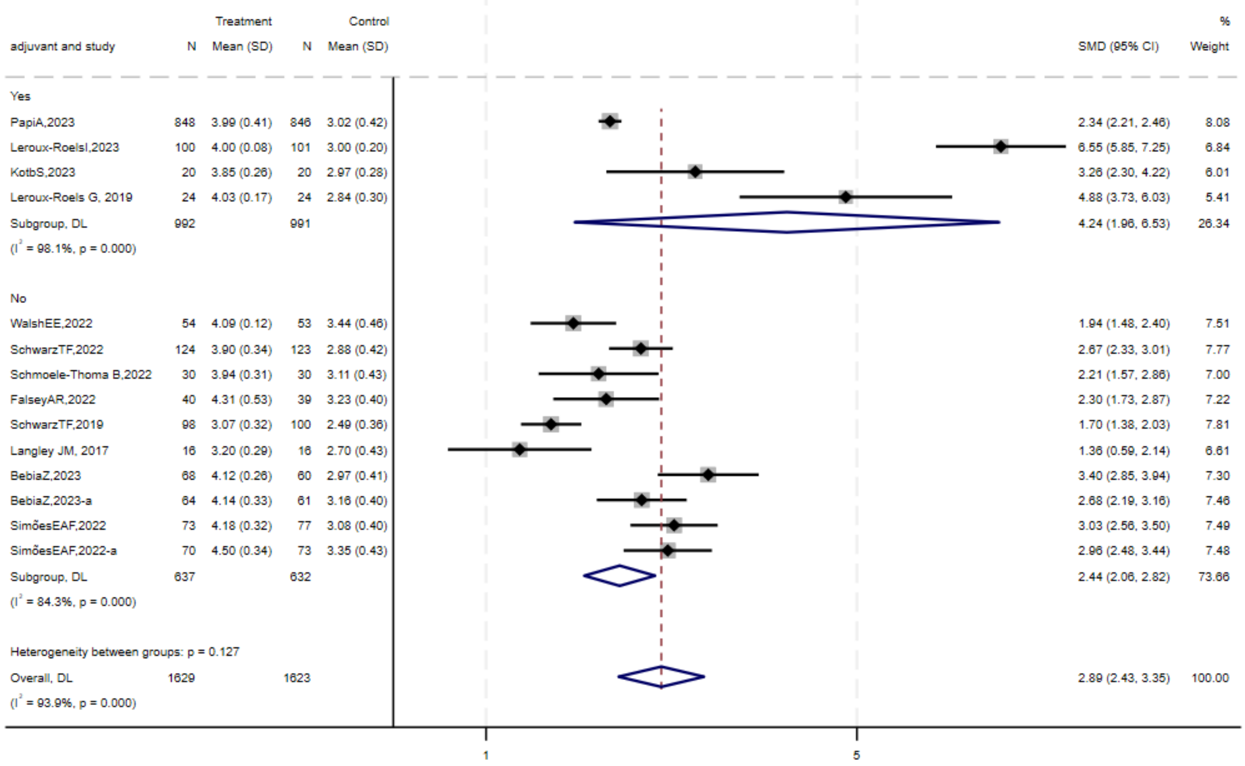

(B)

Figure S6. (**A**) Subgroup analysis for nAb by age. (**B**) Subgroup analysis for nAb by adjuvant. [17,20,22,23,25-32].

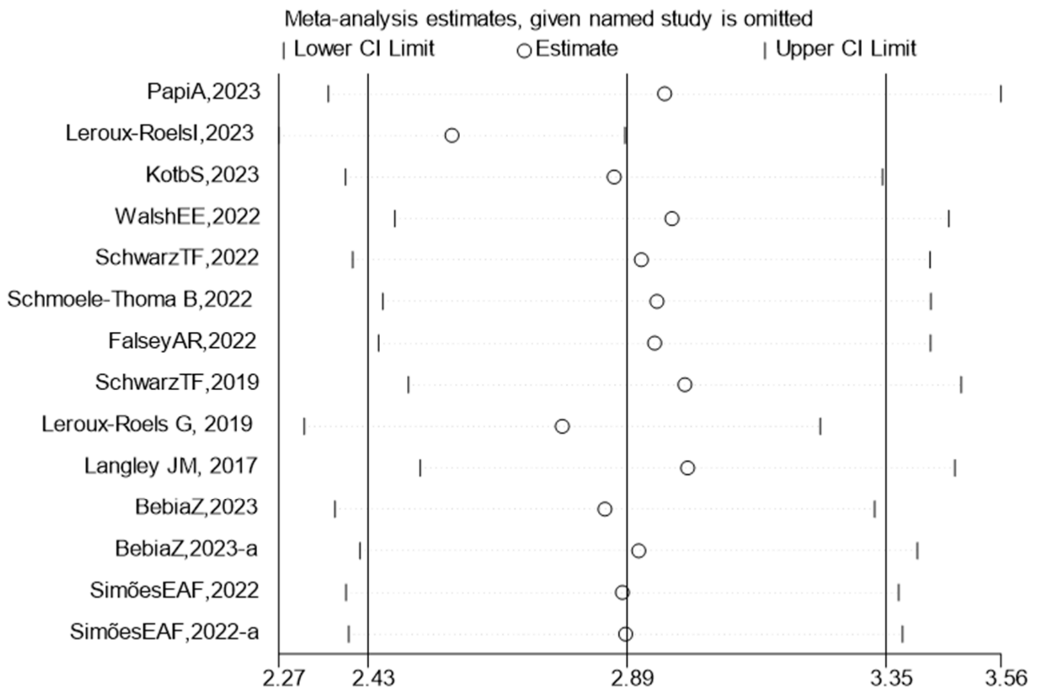

Figure S7. Sensitivity analysis for nAb. [17,20,22-23,25-32].

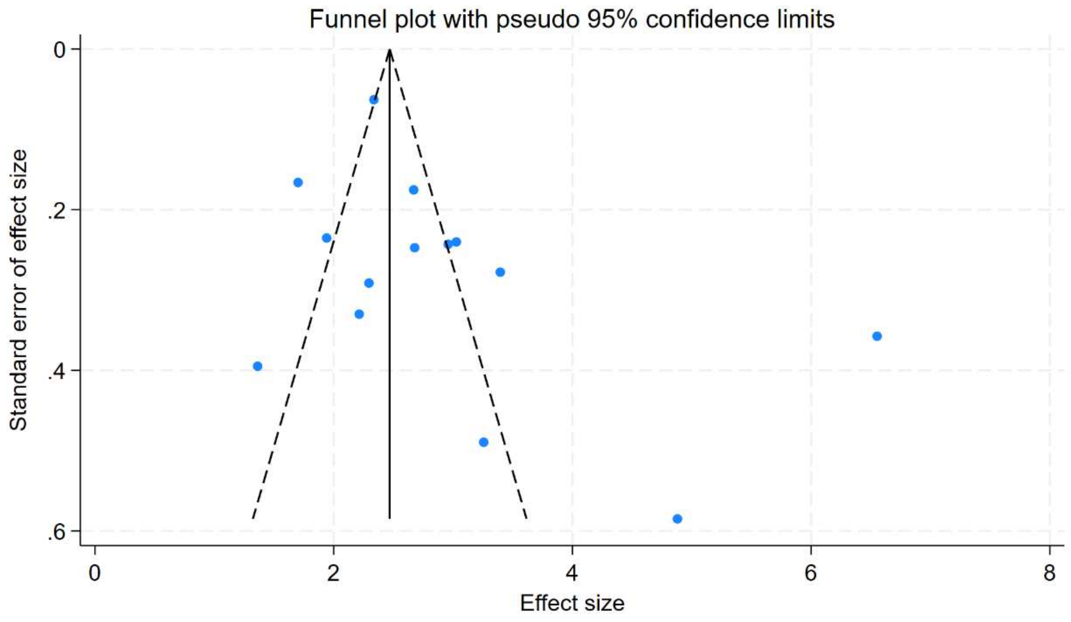

Figure S8. Funnel plot of log-transformed neutralizing antibody. [17,20,22-23,25-32].
